# Factors influencing patient safety in Sweden: perceptions of patient safety officers in the county councils

**DOI:** 10.1186/1472-6963-13-52

**Published:** 2013-02-08

**Authors:** Mikaela Nygren, Kerstin Roback, Annica Öhrn, Hans Rutberg, Mikael Rahmqvist, Per Nilsen

**Affiliations:** 1 Department of Medical and Health Sciences, Division of Health Care Analysis, Linkoping University, 581 83, Linköping, Sweden

**Keywords:** Patient safety, Patient involvement, Communication, Safety culture, Root cause analysis, Risk analysis, Incident reporting

## Abstract

**Background:**

National, regional and local activities to improve patient safety in Sweden have increased over the last decade. There are high ambitions for improved patient safety in Sweden. This study surveyed health care professionals who held key positions in their county council’s patient safety work to investigate their perceptions of the conditions for this work, factors they believe have been most important in reaching the current level of patient safety and factors they believe would be most important for achieving improved patient safety in the future.

**Methods:**

The study population consisted of 218 health care professionals holding strategic positions in patient safety work in Swedish county councils. Using a questionnaire, the following topics were analysed in this study: profession/occupation; number of years involved in a designated task on patient safety issues; knowledge/overview of the county council’s patient safety work; ability to influence this work; conditions for this work; and the importance of various factors for current and future levels of patient safety.

**Results:**

The response rate to the questionnaire was 79%. The conditions that had the highest number of responses in complete agreement were “patients’ involvement is important for patient safety” and “patient safety work has good support from the county council’s management”. Factors that were considered most important for achieving the current level of patient safety were root cause and risk analyses, incident reporting and the Swedish Patient Safety Law. An organizational culture that encourages reporting and avoids blame was considered most important for improved patient safety in the future, closely followed by improved communication between health care practitioners and patients.

**Conclusion:**

Health care professionals with important positions in the Swedish county councils’ patient safety work believe that conditions for this work are somewhat constrained. They attribute the current levels of patient safety to a broad range of factors and believe that many different solutions can contribute to enhanced patient safety in the future, suggesting that this work must be multifactorial.

## Background

Patient safety has progressed in less than a decade from being a relatively insignificant topic to having a position high on the agenda for managers, providers and policymakers in health care as well as the general public. National, regional and local activities to improve patient safety in Sweden have increased markedly since 2008 when a national study [1] on the incidence and nature of adverse events estimated that the percentage of preventable adverse events was as high as 8.6% in Swedish hospital care. Initiated by the National Board of Health and Welfare, the study was important because it clearly demonstrated that the magnitude of the patient safety problem was not smaller in Sweden than elsewhere; the results were comparable with many international studies of adverse events [2-5]. The study led to a stronger emphasis on patient safety issues in Sweden and a considerable increase in activities to achieve improved patient safety in the county councils, which are responsible for the provision of health care to the residents in each county council.

The Swedish Association of Local Authorities and Regions (SALAR), representing the county councils and municipalities, has played a key role in Swedish patient safety efforts. They have organized patient safety conferences, set up networks of experts and policymakers, and published widely distributed handbooks and evidence-based guidelines for health issues such as falls, pressure ulcers, medication errors in health care transitions and health care-associated infections. Sweden generally has strong locally based quality improvement programs and has focused on the relationship between quality and leadership [6]. Efforts for improved patient safety in Sweden were further enhanced in 2011 with the introduction of a new law on patient safety [7] and a government-supported financial incentive plan initiated by SALAR, which has allocated over two billion SEK for 2011–2014 to county councils that carry out certain patient safety-enhancing actions and achieve specific results regarding patient safety. Inspired by Sweden’s long-term road safety goal that there should be no fatalities or serious injuries due to road traffic, a zero vision has been discussed for adverse events in Swedish health care [8].

This high ambition for improved patient safety in Sweden raises the question of how can this be achieved. Efforts for increased patient safety are often complex and multifaceted, targeted at many different levels, including individual health care practitioners, teams, managers and patients, and use many different strategies [9]. Much patient safety work tends to be pragmatic and experience-based rather than relying on solid evidence of effectiveness [10]. As Vincent [11], p. 374 points out, the urge to “get on and change things” often takes precedence over carefully planned and evaluated efforts. These difficulties make it important to investigate the opinions of those in charge of patient safety efforts in Sweden’s 21 county councils: what do they consider the most important activities to attain improved patient safety? This study investigates the perceptions of health care professionals who hold key positions in county council patient safety work on the conditions for this work and factors they believe have been most important in reaching the current level of patient safety, as well as factors they believe would be most important for achieving improved patient safety in the future. These issues have not been investigated previously but are important for analysis of Swedish ambitions for improved patient safety.

## Methods

### Study setting and participants

Health care in Sweden is mainly government-funded and decentralized, although private health care also exists. All residents are insured by the state, with equal access for the entire population. Out-of-pocket fees are low and regulated by law. The provision of health care services in Sweden is primarily the responsibility of the 21 county councils throughout Sweden. The health care system is financed primarily through taxes levied by county councils and municipalities [12].

The study population consisted of 218 health care professionals holding strategic positions in patient safety work in Swedish county councils, which can be considered the meso level of Swedish health care; these professionals are referred to as patient safety officers in this article.

This study population was defined as people within the county councils who (1) had a designated task involving patient safety issues, (2) had knowledge/overview of the county council’s patient safety work, and (3) had the ability to influence this work. The patient safety officers were recruited in collaboration with designated members in a SALAR patient safety network, representing all 21 county councils. These representatives were asked to identify respondents whom they considered had “good knowledge and overview of the county council’s patient safety work and the ability to influence decisions concerning these efforts”. The number of patient safety officers from each county council ranged from 3 to 15, and was proportional to the population size and health care budget of each county council.

Ethical approval was not sought for this study as it did not involve sensitive personal information as specified in the Swedish law regulating the ethical approval for research concerning humans [13].

### The questionnaire

The questionnaire was developed in collaboration with patient safety researchers from the Royal Institute of Technology and policymakers from SALAR and the National Board of Health and Welfare. The selection of questions and response items was based on the literature and discussions among the questionnaire developers [14,15]. Some of the response items on factors associated with patient safety were obtained from two previous studies [14,16]. The questionnaire was reviewed by an expert in respondent psychology and survey methodology. In addition, a cognitive interview was conducted with a person who was familiar with the subject of patient safety from a county council perspective.

The questionnaire consisted of six pages encompassing questions divided into eight sections preceded by a brief introduction explaining why the survey was being undertaken. Questions from seven sections were extracted for use in this study: (1) profession/occupation; (2) number of years involved in a designated task on patient safety issues; (3) knowledge/overview of the county council’s patient safety work (Likert scale, from poor to excellent knowledge/overview); (4) ability to influence this work (Likert scale, from poor to excellent ability); (5) conditions for the county council’s patient safety work (18 response items scored on a Likert scale, from do not agree to agree completely); (6) importance of various factors in attaining the current level of patient safety in the county council (36 response items scored on a Likert scale, from not at all important to very important); and (7) importance of factors to achieve improved patient safety in the future (22 response items, scored on a Likert scale, from not at all important to very important, with an additional option of “cannot be improved further”). Sections 6 and 7 included space for comments and additional items suggested by the respondents.

### Data collection and analysis

The questionnaire was sent to the respondents by mail in October 2011 together with a stamped return envelope. All respondents received two reminders by e-mail 3 and 5 weeks after the first mailing. The second reminder included a PDF version of the questionnaire, which the respondents could print out, fill in and return by regular mail. Data from the questionnaires were entered independently into an MS Office Access database by two persons. A third person examined the database to validate all the data entries. Descriptive statistics were obtained using SPSS. Comments and additional items suggested by the respondents have not been further analysed.

## Results

### Participants and response rate

The questionnaire was sent to 218 patient safety officers. Two officers had resigned from their respective county council and thus did not receive the questionnaire, resulting in a study population of 216 (Table 1). Of these, 171 people answered the questionnaire, yielding a response rate of 79%. Three respondents were excluded from the analysis due to incomplete answers.

**Table 1:** Respondent characteristics

	**%**	***N***
Occupation/profession in the county council*		
Physician		87
Administrative personnel		82
Nurse		17
Other		3
**Time designated to work with patient safety issues**		
<1 year	16	27
1–2 years	19	32
3–5 years	27	45
>5 years	38	62
**Knowledge/overview of the county council’s patient safety work**		
Excellent	20	33
Very good	45	75
Good	30	51
Fair	5	8
Poor		0
**Ability to influence the patient safety work in the county council**		
Excellent	9	15
Very good	33	54
Good	44	72
Fair	13	22
Poor	2	3

Answers missing or recorded as “no opinion” excluded.

*Comments: 19 respondents had more than one answer, one answer was missing.

### Respondent characteristics

Two-thirds of the respondents (65%) had been designated to work with patient safety issues in their county council for 3 years or more (Table 1). Two-thirds (65%) felt that they had excellent or very good knowledge of the county council’s patient safety work, and 42% believed that they had excellent or very good ability to influence the patient safety work in their county council.

### Conditions for the county council’s patient safety work

The conditions that had the highest number of responses in complete agreement were “patients’ involvement is important for patient safety (43%)” and “patient safety work has good support from the county council’s management” (32%)” (Table 2). All other response items had considerably lower rates, ranging from 17 to 1%, of “agree completely” replies. The lowest proportions of “agree completely” replies were: “interventions implemented to improve patient safety are evaluated” (2%); “information about adverse events and risks are systematically communicated to health care personnel throughout the county council” (2%); and “the county council is supportive in committing resources of various kinds” (1%).

**Table 2:** Conditions for the county council’s patient safety work

**Response items**	**To what extent do the following statements apply to the county council in which you work?**				***N***
	**Agree completely (%)**	**Agree mostly (%)**	**Agree slightly (%)**	**Do not agree at all (%)**	
Patient involvement is important for patient safety	43	41	14	3	164
Patient safety work has good support from the county council’s management	32	47	21	0	167
Patient safety work has good support from the heads of departments/clinics	17	58	25	1	166
Inadequate time and/or resources to analyse adverse events and risks	13	53	31	3	167
Inadequate time and/or resources for preventive action	12	61	23	3	163
The county council has good systems in place for conducting root cause analyses	12	59	22	2	166
The county council provides a supportive environment for patient safety work	11	55	29	5	167
The county council has good systems in place for analysing adverse events	11	48	31	10	164
The county council provides support for forums and meetings concerning patient safety	11	40	41	8	165
The county council has good systems in place for conducting risk analyses	11	38	40	9	166
Personnel who work with patient safety are required to have specific training in the area	6	32	48	13	162
Root cause and risk analyses and related analyses result in changes to routines and practice	5	60	33	1	162
Patient complaints and reports are systematically analysed and followed up	5	52	41	2	164
Identification of adverse events and risks usually results in interventions to improve patient safety	4	50	43	4	161
There is an adequate budget for patient safety work	2	26	42	30	156
The interventions implemented to improve patient safety are evaluated	2	23	64	11	160
Information about adverse events and risks are systematically communicated to health care workers throughout the county council	2	21	54	24	165
The county council is supportive and commits resources of various kinds (financial support, personnel, etc.) to improve patient safety	1	15	55	29	161

Response items are arranged according to the proportion of “agree completely” replies. Answers missing or recorded as “no opinion” excluded.

### Factors involved in attaining the current level of patient safety

More than half of the respondents considered seven factors as “very important” for the current level of patient safety in the county council (Table 3). Factors that were rated highest included “conducting root cause and risk analyses” (66%), “incident reporting” (63%) and “the Swedish Patient Safety Law” (60%). Approximately one-third of the respondents believed the use of various SALAR guidelines were very important for attaining the current level of patient safety. Relatively few of the respondents identified “structured review of medical records” (30%), “patient safety culture surveys” (26%), “information from various quality registers” (26%) and “research and scientific articles about patient safety” (19%) as very important factors.

**Table 3:** Factors to attain the current level of patient safety

**Response items**	**How important have the following 36 factors been to achieve the current level of patient safety in your county council?**				***N***
	**Very important (%)**	**Moderately important (%)**	**Slightly important (%)**	**Not at all important (%)**	
Conducting root cause and risk analyses	66	32	2	0	168
Incident reporting	63	33	4	0	165
The Swedish Patient Safety Law	60	30	8	2	165
Internal discussions with county council management, heads of health care units, health care providers etc.	57	35	7	1	162
Efforts to reduce the use of antibiotics	56	40	3	1	159
Use of Safe Surgery checklist	54	42	4	0	127
PPM of adherence to hygiene rules	51	41	8	0	161
Participation in SALAR's PPM of HAI	49	44	6	1	156
Use of *Handbook for Patient Safety Work: Risk Analysis and Root Cause Analysis*	48	41	10	1	155
Swedish regulation: SOSFS 2005:12, Quality and patient safety in health care, as described in the handbook *Good Care*	45	41	10	3	164
Participation in SALAR's PPM of compliance with hygiene rules	43	45	11	1	159
Surveillance of pressure ulcers	42	46	11	0	157
Participation in SALAR’s PPM of pressure ulcer	41	50	9	1	153
Legal decision from Lex Maria cases	39	50	11	0	165
Local STRAMA group	36	54	9	2	160
Use of SALAR's guidelines on postoperative infections	35	46	18	1	142
Complaints and reports from patients	34	51	13	2	163
Use of SALAR's guidelines on falls and injuries from falls	34	51	15	1	144
Use of SALAR's guidelines on pressure ulcers	34	48	18	1	143
Use of SALAR's guidelines on hospital acquired urinary tract infections	34	47	18	1	146
Use of SALAR's guidelines on malnutrition	33	39	26	2	141
Use of SALAR's guidelines on medication errors in health care transitions	32	45	22	1	148
Use of SALAR's guidelines on infections of central venous catheter	32	47	19	1	142
Use of SALAR's guidelines on medication-related problems	31	57	11	2	159
External discussions with others involved in patient safety	31	49	20	1	147
Structured review of medical records	30	33	28	10	144
Patient safety culture surveys	26	48	19	7	155
Information from various quality registers	26	42	27	4	157
Assembling annual report of patient safety work in the county council	20	42	29	9	157
Research and scientific articles about patient safety	19	42	36	3	162
Use of *Handbook for Patient Safety: Structured Review of Medical Records According to Global Trigger Tool*	18	37	30	15	131
Participation in the national patient survey in primary health care	17	45	32	5	115
Use of *Handbook for Patient Safety: How to Measure Patient Safety Culture*	17	46	32	6	145
Participation in the National Patient Overview (medical records at the national level)	15	42	31	12	121
Use of *Handbook for Patient Safety: Safer Care*	12	42	34	12	113
Informational material from the County Council Mutual Insurance Company	11	55	29	5	158

Answers missing or recorded as “no opinion” excluded.

Abbreviations: HAI, health care-associated infection; Lex Maria, regulation in Sweden that obliges caregivers to report incidents that have resulted or could have led to serious health damage to the National Board in Sweden; PPM, point prevalence measurement; SALAR, Swedish Association of Local Authorities and Regions; STRAMA, the Swedish strategic programme against antibiotic resistance.

### Factors for achieving improved patient safety in the future

Six factors were considered “very important” for achieving improved patient safety in the future by more than 60% of the respondents (Table 4). The highest proportions were noted for “improvement of organizational culture that encourages reporting and avoids blame” (83%), “improved communication between health care practitioners and patients” (80%) and “improved communication among health care practitioners” (78%). An increase in the number of physicians and nurses was only considered to be “very important” by 24% and 20%, respectively.

**Table 4:** Factors for achieving improved patient safety in the future

**Response items**	**Based on the current level of patient safety in your county council, how important do you think the following 22 factors would be toachieve increased patient safety?**					***N***
	**Very important (%)**	**Moderately important (%)**	**Slightly important (%)**	**Not at all important (%)**	**Cannot be further improved (%)**	
Improvement in organizational culture to encourage reporting and avoid blame	83	22	1	0	0	167
Improved communication between health care practitioners and patients	80	39	1	0	0	167
Improved communication among health care practitioners	78	16	1	0	0	167
Incorporation of patient safety education as a compulsory component of basic education for health care practitioners	77	19	1	0	1	167
Improved infection control, including improved hand hygiene	64	21	2	0	0	165
Increased education/training in issues related to patient safety for health care practitioners	60	31	5	0	1	165
Increased standardization of medical technology equipment and products	59	42	5	0	1	166
Improved logistics concerning hospital beds and overcrowding	52	39	7	0	2	166
Improved instruction/training concerning medical equipment	52	31	8	0	2	166
Stronger control from top-level management	50	36	11	1	1	167
Increased involvement by pharmacists, such as at hospital rounds	30	52	14	2	2	166
Increased number of physicians	24	60	24	1	2	166
More guidelines and recommended actions to guide the work of patient safety	22	50	23	4	1	166
Increased legal requirement to carry out activities and achieve results in terms of patient safety	22	44	27	9	2	166
Increased number of nurses	20	45	30	7	6	163
Continued financial incentive plan for the implementation of activities, achievements, etc.	19	35	33	8	2	166
More hospital beds	17	38	36	8	1	164
Confidential reporting of adverse events and incidents to an independent authority	15	29	40	7	4	163
Reduced penalty for staff who make mistakes	14	22	42	8	4	163
Increased collaboration with researchers	13	27	42	13	4	165
Reduced working hours for physicians	12	21	43	17	4	161
Increased penalty for personnel who make mistakes	2	6	38	49	6	159

Response items arranged according to the proportion of “very important” replies.

## Discussion

This study showed that health care professionals with key positions in Swedish county councils’ patient safety work attributed the current level of patient safety to a broad range of factors and believed that many different interventions, practices and approaches could contribute to improved patient safety, thus emphasizing the importance of multifactorial solutions to the patient safety problem. However, the conditions for patient safety work seemed to have plenty of room for improvement according to the patient safety officers.

The respondents stated to a large extent that patient involvement is important for patient safety. There is an international trend towards greater patient involvement in health care delivery [17], but there is still a paucity of research findings on the acceptability to patients of a new patient role and the extent to which such involvement actually leads to safety improvements [18]. Research has identified numerous barriers to enlisting patients in efforts to improve patient safety including limited acceptance of a more active patient role [19] and insufficient health literacy, i.e. the capacity to obtain, process and understand basic health information and services needed to make appropriate health decisions [20]. The fact that we observed limited agreement with the statement “patient complaints and reports are systematically analysed and followed up” suggests that it is easier to profess that patient involvement is important than to develop a systematic strategy that utilizes information from patients. There have been calls for more research for better understanding of how patients can be involved in their own care [18,21-23].

Incident reporting and conducting root cause and risk analyses were identified as the most important factors for achieving the current levels of patient safety. These findings are in line with a Dutch survey of primary care physicians and researchers from eight countries, which found that reporting and analysis of incidents was considered very important [15]. Local reporting systems have been given a dominant role in the drive to improve patient safety in Sweden [24]. All Swedish county councils have computerized reporting systems and any health care practitioners can submit incidents [25]. However, the reliance on incident reporting systems in many countries has been questioned by international researchers who claim that these systems are insufficient on their own to identify incidents and need to be supplemented with other information from patients and retrospective chart reviews [26-30]. It has been suggested that more process-oriented, rather than outcome-oriented, information is required to obtain a more complete picture of incidents and promote a blame-free reporting culture [31]. Another important issue is the extent to which patient safety-related data are analysed, and how this may trigger appropriate actions and lead to organizational learning. Research on how data are transformed into appropriate strategies and learning is needed.

The respondents expressed conviction that an improved organizational culture that encourages reporting and avoids blame can result in enhanced patient safety. There has been a strong focus on patient safety culture in patient safety research and policymaking in the last decade, but relatively few studies [32-35] have actually demonstrated a positive relationship between this culture and outcomes. Although the respondents were convinced of the importance of an improved organizational culture that avoids blame and shame, researchers have highlighted the complexity of the culture concept as we do not know what aspects of the patient safety culture are most in need of improvement and how and whether these can be accomplished [36]. Despite the importance attributed to patient safety culture, the use of the *Handbook for Patient Safety – How to Measure Patient Safety Culture* (one of the handbooks distributed by SALAR) was considered to have played a minor role in achieving the current level of patient safety. All Swedish county councils conduct patient safety culture measurements, but these have only been carried out for a few years so it is unlikely that they have had any influence on the culture as yet. Research is needed to examine how results from culture assessments can be fed back to health care practitioners at the micro, meso and macro levels of health care and how various strategies can be selected and implemented on the basis of the results of such assessments.

Communication was also identified as a critical factor for achieving enhanced patient safety in our study, both improved communication among health care practitioners themselves and between practitioners and patients. The concepts of communication and culture overlap because an open communication based on mutual trust is considered an integral aspect of a beneficial patient safety culture [11]. Communication is usually measured as part of patient safety culture assessments. Instruments such as the *Stanford PSC Survey*[36], the *Manchester Patient Safety Framework* and *AHRQ’s Hospital Survey on Patient Safety Culture*[37] all incorporate questions on communication.

Patient safety-related training and education was identified as another important factor to achieve improved patient safety. Patient safety is not a compulsory subject in the basic education of physicians and nurses in Sweden. Clinical training in Sweden, much like elsewhere, is typically organized around basic science themes, body systems or core specialty competencies. Hence, there are no courses for Swedish health care professionals that focus specifically on patient safety matters. Specific and more general patient safety-related knowledge must be acquired through participation in continuing professional education, with courses being offered at some universities in Sweden. However, these tend to be costly and reach small numbers of health care practitioners. Öhrn [38] has argued that more education and training in many patient safety issues is needed to increase Swedish health care practitioners’ knowledge and understanding of patient safety problems and to facilitate the development of more high-reliability health care organizations. Research on patient safety-related education and training has predominantly focused on targeted issues such as teamwork or simulation training, with far less attention given to activities aimed at increasing awareness and knowledge of patient safety issues more generally.

Our findings on the importance of achieving a blame-free organizational culture that encourages reporting, improved communication between staff and patients, as well as better education and training are very similar to those of a study of health care leaders undertaken in 2005 in Sweden [14]. The previous Swedish study identified these three areas as the most important to attain improved patient safety. Similar findings were also noted in a Dutch survey of primary care physicians and researchers, where factors such as “measurement and feedback on safety culture in general practices”, “culture and mentality which facilitates learning from incidents” and various aspects related to education and training in patient safety-related issues were among the factors considered most important for patient safety [15].

The respondents in our study did not consider that workforce issues, such as reduced working hours for physicians or increased numbers of physicians or nurses, were important in order to achieve improved patient safety. These findings contrast somewhat with those in a study conducted in the United States by Blendon *et al*. [16], which identified increased numbers of nurses in hospitals, more time for physicians to spend with patients and reduced working hours for physicians in training as very important factors in achieving enhanced patient safety. However, their study population consisted of practicing physicians and members of the public. The respondents in our study were not frontline health care practitioners, which may provide a partial explanation for their low rating of these workforce issues. It would seem self-evident that a reduction in working hours should lead to improvements in patient safety. There is strong evidence that fatigue impairs clinical performance, but a simple mandate of working fewer hours may not yield improved patient care for many reasons [39,40].

Some of our findings imply that patient safety work in Sweden is largely experience-based rather than evidence-based. For instance, few respondents felt that “research and scientific articles about patient safety” or “increased collaboration with researchers” were important. The role of research and evidence in patient safety practices is debated among patient safety researchers. Those who believe that patient safety work is too complex to study with scientific rigour argue that many practices have little downside and should be implemented when improvements can be expected, whereas other researchers hold that practices should be studied to the extent possible even if experimental research conditions are difficult to achieve [41]. The use of many of the guidelines produced by SALAR (e.g. *Postoperative Infections*, *Falls and Injuries from Falls*, *Malnutrition*), were perceived to have been important factors in reaching the current levels of patient safety. These guidelines consist of recommendations to achieve safer health care and are widely disseminated to Swedish health care practitioners for use at the micro level of health care. They are based on the latest research findings assembled by expert panels consisting of researchers and meso- and micro-level health care practitioners; key results and conclusions from research are summarized and presented in formats that make them easy to digest. The use of these guidelines suggests that research has an important role in Swedish patient safety work but also indicates that research must be summarized and presented in abbreviated form to be relevant for busy practitioners at the sharp end of health care.

Somewhat surprisingly, the new Patient Safety Act was considered very important for today’s patient safety levels. The law is so new that it cannot have affected the county councils’ patient safety work. However, the law appears to have served an important function in raising awareness of the importance of the patient safety issue, thus providing crucial support for the initiatives taken by the patient safety officers at the meso level of Swedish health care. The impact of the law among health care practitioners at the micro level is currently not known.

This study has some shortcomings that must be considered when interpreting the results. The survey questionnaire has not been validated in research studies, but it was partially based on existing questionnaires described in the literature [14,16]. Furthermore, the questionnaire underwent a thorough development process (lasting 6 months) to ensure that its content, structure and the formulation and wording of the individual questions would work well for the respondents. The content of three sections (conditions for patient safety work, factors of importance for attaining the current levels of patient safety and for achieving enhanced patient safety in the future) were discussed with many of the leading and most experienced Swedish patient safety researchers and representatives from SALAR. The response rate was quite high at 79%, but nevertheless provides some scope for response bias. Non-responders in survey research are usually quite different from those who participate, thus limiting the investigator’s ability to make generalizations about the entire population. Social desirability bias may have served to produce more positive accounts of patient safety issues than are actually the case. However, the questions generally did not concern the respondents’ attitudes or opinions concerning patient safety, but rather investigated their perceptions of various conditions for the county councils’ patient safety work and what factors they believed affected patient safety.

This study also has considerable strengths. We were able to reach the targeted study population, as most of the respondents believed that they had good knowledge of the county council’s patient safety work and the ability to influence this work. The results provide important knowledge about current patient safety work in Sweden and give an indication of how this work may be further developed.

## Conclusions

Health care professionals with important positions in patient safety work in Sweden’s county councils believe that conditions for this work are somewhat constrained. They attribute the current levels of patient safety to a broad range of factors and believe that many different solutions can contribute to enhanced patient safety in the future, suggesting that this work must be multifactorial. The findings point to several knowledge gaps that require more research and development work, e.g. how patient involvement can contribute to improved patient safety, how data generated in incident reporting systems can be transformed into action and learning, and how patient safety culture assessments can be linked to strategies and improvements in various outcomes. Further research is also needed to investigate the perceptions of health care professionals working at the sharp end of health care of the factors that contribute to improved patient safety.

## Competing interests

The authors report no conflicts of interest. The authors alone are responsible for the content and writing of the paper.

## Authors’ contributions

All authors participated in designing the study and constructing the questionnaire. MN collected the data and drafted the manuscript. KR and PN helped with drafting of the manuscript. MR performed the statistical analyses. All authors read and contributed to the manuscript and approved the final manuscript.

## Pre-publication history

The pre-publication history for this paper can be accessed here:

http://www.biomedcentral.com/1472-6963/13/52/prepub
